# Mast Cell Cytokines in Acute and Chronic Gingival Tissue Inflammation: Role of IL-33 and IL-37

**DOI:** 10.3390/ijms232113242

**Published:** 2022-10-31

**Authors:** Matteo Trimarchi, Dorina Lauritano, Gianpaolo Ronconi, Alessandro Caraffa, Carla E. Gallenga, Ilias Frydas, Spyros K. Kritas, Vittorio Calvisi, Pio Conti

**Affiliations:** 1 Centre of Neuroscience of Milan, Department of Medicine and Surgery, University of Milan, 20122 Milano, Italy; trimarchi.matteo@hsr.it; 2 Department of Translational Medicine, University of Ferrara, 44121 Ferrara, Italy; dorina.lauritano@icloud.com; 3 Clinica dei Pazienti del Territorio, Fondazione Policlinico Gemelli, 00185 Rome, Italy; gianpaolo.ronconi@policlinicogemelli.it; 4 School of Pharmacy, University of Camerino, 62032 Camerino, Italy; alecaraffa@libero.it; 5 Section of Ophthalmology, Department of Biomedical Sciences and Specialist Surgery, University of Ferrara, 44121 Ferrara, Italy; gllcln@unife.it; 6 Department of Parasitology, Aristotle University, 54124 Thessaloniki, Greece; ilias.frydas@gmail.com; 7 Department of Microbiology and Infectious Diseases, School of Veterinary Medicine, Aristotle University of Thessaloniki, 54124 Macedonia, Greece; skrh46@gmail.com; 8 Orthopaedics Department, University of L’Aquila, 67100 L’Aquila, Italy; vittorio.calvisi@univaq.it; 9 Immunology Division, Postgraduate Medical School, University of Chieti, 65100 Pescara, Italy

**Keywords:** mast cell, inflammation, autoimmunity, cytokine, periodontal disease, periodontitis, gingivitis, ST2 receptor

## Abstract

Much evidence suggests autoimmunity in the etiopathogenesis of periodontal disease. In fact, in periodontitis, there is antibody production against collagen, DNA, and IgG, as well as increased IgA expression, T cell dysfunction, high expression of class II MHC molecules on the surface of gingival epithelial cells in inflamed tissues, activation of NK cells, and the generation of antibodies against the azurophil granules of polymorphonuclear leukocytes. In general, direct activation of autoreactive immune cells and production of TNF can activate neutrophils to release pro-inflammatory enzymes with tissue damage in the gingiva. Gingival inflammation and, in the most serious cases, periodontitis, are mainly due to the dysbiosis of the commensal oral microbiota that triggers the immune system. This inflammatory pathological state can affect the periodontal ligament, bone, and the entire gingival tissue. Oral tolerance can be abrogated by some cytokines produced by epithelial cells and activated immune cells, including mast cells (MCs). Periodontal cells and inflammatory–immune cells, including mast cells (MCs), produce cytokines and chemokines, mediating local inflammation of the gingival, along with destruction of the periodontal ligament and alveolar bone. Immune-cell activation and recruitment can be induced by inflammatory cytokines, such as IL-1, TNF, IL-33, and bacterial products, including lipopolysaccharide (LPS). IL-1 and IL-33 are pleiotropic cytokines from members of the IL-1 family, which mediate inflammation of MCs and contribute to many key features of periodontitis and other inflammatory disorders. IL-33 activates several immune cells, including lymphocytes, Th2 cells, and MCs in both innate and acquired immunological diseases. The classic therapies for periodontitis include non-surgical periodontal treatment, surgery, antibiotics, anti-inflammatory drugs, and surgery, which have been only partially effective. Recently, a natural cytokine, IL-37, a member of the IL-1 family and a suppressor of IL-1b, has received considerable attention for the treatment of inflammatory diseases. In this article, we report that IL-37 may be an important and effective therapeutic cytokine that may inhibit periodontal inflammation. The purpose of this paper is to study the relationship between MCs, IL-1, IL-33, and IL-37 inhibition in acute and chronic inflamed gingival tissue.

## 1. Introduction

The immune system includes molecular and cellular elements that defend our organism which do not react against self-proteins (immune tolerance) [1]. It is well-known that loss of tolerance leads to autoimmune disease. More than 50 years ago, Brandtzaeg and Kraus described the autoimmune basis in the pathogenesis of periodontal disease, where microorganisms can establish or influence the process of autoimmunity [2].

Microbiota are microorganisms that live in symbiosis in the body and are found in the gastric and mouth apparatus [3]. The modified microbiome may play an important role in oral flora, in periodontal and intestinal disease, and in the pathogenesis of autoimmune diseases [4]. Therefore, there is a symbiotic relationship between the host and its microorganisms. In fact, commensal bacteria protect against inflammatory diseases and allergies [5]. Chronic inflammatory disease has a multifactorial etiology and can affect the oral and gastrointestinal systems, leading to dysbiosis, an alteration of the balance of the bacterial flora with consequent disturbances and change of the microbiota and innate immune system [6]. It has recently been reported in the literature that periodontitis is a chronic immune and inflammatory disease, with its primary etiology being bacterial plaque [7]. The interaction between the microbiome and the innate immune system can occur in many different diseases. The microbiota is important for tissue health, and its alteration can lead to chronic and autoimmune diseases, as microbial products can continuously stimulate innate immune responses and lead to chronic inflammation [8]. Therefore, continued self-inflammation due to altered host–microbiota and the innate immunity axis can lead to autoimmune disease.

Much evidence leads to autoimmunity in the etiopathogenesis of periodontal disease, with the production of antibodies against collagen, DNA, and IgG; increased IgA expression; dysfunction of T-helper or T-suppressor cells; activation of NK cells, pro-inflammatory cytokine secretion; and increased expression of class II MHC molecules on the surface of gingival epithelial cells in inflamed tissues [9]. In addition, in periodontitis, antibodies against the azurophil granules of polymorphonuclear leukocytes can be detected [10]. Direct activation of T-cell-mediated autoreactive B cells and the production of TNF by mast cells (MCs) can activate neutrophils to release pro-inflammatory enzymes with cell damage in the gingival tissue [11]. Gingivitis is the mildest form of plaque-induced periodontal disease (PD), while periodontitis is a more severe, chronic immune and inflammatory disease, with the primary etiology being bacterial plaque. Infectious inflammatory diseases of the gum are characterized by local redness, edema, and bleeding of the periodontal tissues following the accumulation of bacterial plaque [12]. If the disease is left untreated, destruction of the periodontal attachment apparatus may occur, which can lead to the loss of connective tissue, tooth mobility, and tooth loss [13,14].

Gingival inflammation and, in the most serious cases, periodontitis, are mainly due to the dysbiosis of the commensal oral microbiota that triggers the immune system [15]. This inflammatory pathological state can affect the periodontal ligament, bone, and the entire gingival tissue. MCs are a potential source of cytokines and chemokines, as seen in mouse and human cell experiments performed in vitro [16]. In periodontal disease, pathogens trigger the immune response mediated by various inflammatory compounds, including cytokines and chemokines, causing bleeding, swelling, bad breath, destruction of gingival tissue, and tooth loss [17].

Gingival inflammation can lead to periodontal disease, where cytokines activate and stimulate MCs to secrete pro-inflammatory molecules that participate in the pathological state of the tissue and, therefore, play a critical role in the induction of inflammation [18]. Elevated levels of pro-inflammatory cytokines, such as IL-1, TNF, and IL-6, are secreted by various immune cells, including MCs. The role of MCs in periodontal disease is not yet clear; however, in this pathology, MCs increase in number, as well as the production of inflammatory cytokines, thus demonstrating their involvement in the resorption of alveolar bone. In the oral tissue, MCs producing cytokines and proteases (tryptase and chymase) promote the infiltration of leukocytes, causing the degradation of the extracellular matrix and leading to gingivitis and periodontitis [19]. In acute inflammation, MCs release various pro-inflammatory molecules, such as histamine, proteoglycans, metabolites of arachidonic acid, TNF, and tryptase, a serine proteinase that promotes inflammation. Histamine, acting on the endothelium, mediates vascular permeability and promotes adhesion of platelets through the adhesion molecule P-selectin.

MCs participate in the acute inflammatory type-1 hypersensitivity reaction, which can occur within minutes. This reaction is mediated by IgE, which binds to the FcεRI receptor of MCs and leads to the release of newly synthesized mediators with tissue alterations and leukocyte activations [16].

In gingival tissue, repeated persistent stimuli on tissue MCs can lead to a chronic inflammatory reaction or late-phase reaction, mediated by innate immune cells with consequent tissue alteration [20]. Cytokine secretion through the FcεRI receptor activates MCs in mice, which can be redundant with other cell types and can cause chronic tissue damage. Activation of the FcεRI and FcγRs receptors of MCs can cause the release of various pro-inflammatory compounds, including IL-1, which contributes to chronic inflammation (as, for example, occurs in rheumatoid arthritis) [16].

Periodontal cells and inflammatory–immune cells, including MCs, produce cytokines and chemokines, mediating local inflammation of the gingival tissue, along with destruction of the periodontal ligament and alveolar bone [15]. Immune-cell activation and recruitment can be induced by inflammatory cytokines, such as IL-1, TNF, and IL-33; and bacterial products, including lipopolysaccharides (LPS). IL-1 and IL-33 are pleiotropic cytokines from members of the IL-1 family that mediate the inflammation of the MCs and contribute to many key features of periodontitis and other inflammatory disorders [19].

Different ligands activate the respective MC receptors, causing activation, migration, or inhibition. In chronic inflammation, MCs intervene by contributing to tissue repair and remodeling; however, by producing pro-inflammatory cytokines, they damage the tissue, as occurs in periodontal disease [18].

The production of cytokines induces molecules that attract the migration of immune cells. Among these are the chemokines, which are responsible for the selective recruitment of leukocytes and play an important role in periodontal inflammation [20].

CXCL8 is a chemokine produced by PMNs and fibroblasts that increases in inflamed periodontal tissue, recruits inflammatory cells, and cooperates with the CCL2/MCP-1 chemokine, which mainly attracts monocytes/macrophages [20]. Cytokine IL-33 activates several immune cells, including lymphocytes, Th2 cells, and MCs in both innate and acquired immunological diseases [21]. The classic therapies for periodontitis include antibiotics, anti-inflammatory drugs, and surgery, which have been only partially effective. It is general knowledge that a natural cytokine, IL-37, a member of the IL-1 family and a suppressor of IL-1, has received considerable attention for the treatment of inflammatory diseases [21].

Dental bacterial biofilms induce inflammation and are the primary etiological factors of periodontal diseases. Several microbiological studies have shown that the pathogenesis of periodontal disease is mediated by an overgrowth of specific Gram-negative anaerobic bacteria in the gingival tissues. More specifically, *Porphyromonas gingivalis, Treponema denticola*, and *Tannerella forsythia*, known as the *red complex*, and their virulence factors can induce severe inflammation and the destruction of periodontal tissues [22]. Although bacteria are the triggering agents, host defense mechanisms within the periodontal tissues seem to be responsible for most of the tissue damage and for the progression of periodontal diseases.

The innate immune system mounts non-specific responses to the bacterial challenge, including secretion of vasoactive substances, such as histamine and vascular endothelial growth factor (VEGF), which are synthesized by mast cells and other immune cells in the periodontium. In response to a microbial challenge, the periodontal tissues are infiltrated by many phagocytic cells that amplify a cascade of cellular and biochemical events by secreting different inflammatory mediators, including cytokines, chemokines, and arachidonic acid metabolites [19]. Once in the gingival tissues, the polymorphonuclear leukocytes (PMNs) either phagocytose the bacteria that is present or die by apoptosis. In both cases, they release their lysosomal contents, such as elastases and collagenases, into the gingival tissues that contribute to the local destruction of connective tissues. Th1 cells are involved in the first phase of periodontitis; they release pro-inflammatory cytokines, such as IL-1, TNF, and IL-6. Meanwhile, in the late second phase, Th2 cells produce IL-4, which helps the production of antibodies and IL-10, which attempts to inhibit the inflammatory cytokines produced by Th1 cells.

A critical pathway in the initiation of the immune response in the periodontium is the recognition of lipopolysaccharides (LPS), derived from Gram-negative bacteria, by Toll-like receptors (TLRs). TLR activation stimulates an intracellular signaling cascade, leading to the synthesis of pro-inflammatory mediators, including TNF and IL-1, leukocyte migration, and osteoclastogenesis [20], inducing bone loss and progression into periodontitis. Pro-inflammatory mediators stimulate the Langerhans cells, which function as antigen-presenting cells (APCs), to migrate to regional lymph nodes and initiate antigen-specific T-cell proliferation, activating the adaptive immune system.

Several environmental risk factors, including smoking, diabetes mellitus, and psychological stress, may modify the host response and, hence, disease progression, severity, and outcome of periodontal treatment [21].

## 2. IL-1Beta (IL-1β)

IL-1 plays an important role in immunity and inflammation [23]. The IL-1 family includes pro-inflammatory cytokines (IL-1 alpha; IL-1 beta; IL-18; IL-33; and IL-36 alpha, beta, and gamma), receptor antagonist cytokines (IL-1RA, IL- 36-RA, and IL-38), and a cytokine with anti-inflammatory power (IL-37). IL-1 has been localized in the cytoplasm, is proteolytically activated by caspase-1, and is secreted by macrophage-type cells [21]. IL-1, regulated by several proteins, including inflammasomes, is a major pro-inflammatory cytokine in the pathogenesis of periodontal disease.

**Figure 1:**
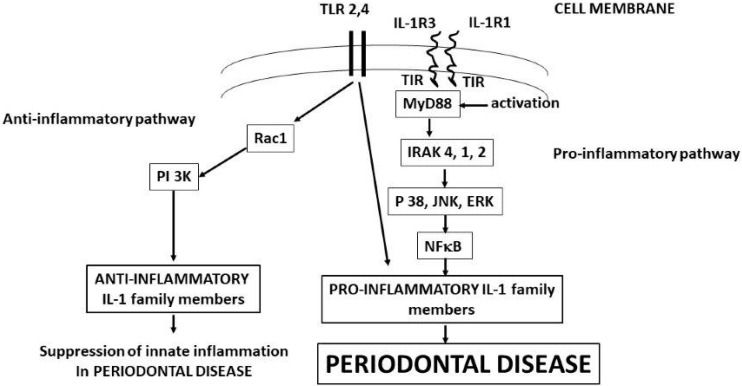
IL-1b binds IL-1R1 and IL-1R3 with the generation of MyD88, starting the biochemical pathways that produces the pro-inflammatory and anti-inflammatory IL-1 family member cytokines that mediate the periodontal disease. Activation of IL-1 precursors leads to periodontal disease, while TLR2 and 4 can lead to both periodontal disease and tissue protection.

Caspase-1 is a cytoplasmatic protease that is activated by the inflammasome complex (Nod-like receptor pyrin domain containing protein 3 (NLRP3) and apoptosis-associated speck-like protein containing CARD (ASC)) [24,25]. Therefore, cytoplasmic pro-IL-1 is the inactive precursor of IL-1 beta, which is proteolytically activated through cleavage by caspase-1, resulting from NLRP3-activated pro-caspase inflammasome [23,24]. The generation of NLRP3 occurs through the pathogen-associated molecular patterns (PAMPs) that induce the transcription factor NF-κB, which activates the inflammasome and IL-1beta precursor genes (Figure 1). NLRP3 inflammasome contributes to the inflammatory state, and its inhibition is considered a potential therapy; however, in several inflammatory diseases, the inhibitory effect has been disappointing [25]. NLRP3 is not only pro-inflammatory but may also mediate beneficial protective signaling (although most of the literature is devoted to its harmful effects). NLRP3 deficiency can lead to a lack of tissue protection by facilitating the activation of TLR2 [25].

IL-1β is known to be involved in the regulation of the innate immune response [13] and is mandatory for auto-inflammatory diseases [26,27,28,29]. We focused on the study of IL-1 beta, because this cytokine promotes inflammatory osteolysis as occurs in chronic periodontitis and other systemic diseases [30]. IL-1β could also be important in diseases involving mast cells and SP [31,32]. IL-1β may also be involved in multiple other diseases that involve MCs, including asthma [33], rheumatoid arthritis [34], multiple sclerosis [35], and psoriasis [36,37,38]. In fact, gene expression and activity of caspase-1 were reported to be increased in lesional psoriatic epidermis [39].

Another activation system may be due to reactive oxygen species (ROS), to the efflux of potassium ions, or to the influx of calcium ions, involving the ROS [33,34]. These effects begin with the recruitment of the Speck-like protein adapter associated with apoptosis containing a CARD (ASC), which, together with the inflammasome and pro-caspase, participate in the active reaction of pro-caspase-1 in caspase-1. At this point, activated caspase-1 can intracellularly cleave pro-IL-1 beta into mature IL-1 beta [35,38] (Figure 1). MCs contain beta pro-IL-1 and caspase-1 and therefore are able to respond to external insults [34] by producing IL-1.

MCs are known to be activated by non-allergic triggers, such as cytokines, but they can also release pro-inflammatory cytokines, such as IL-1 and IL-6, without degranulating [39,40]. In periodontitis, in spite of classical (non-surgical) therapies that include antibiotics and anti-inflammatory drugs, which are often not effective, it is pertinent to think that different anti-IL-1βs, such as soluble IL-1R, IL -1RA, and IL-37, could be used in the clinical therapy for these inflammatory diseases [41,42,43].

## 3. Interleukin-33 (IL-33)

Periodontitis is an inflammatory disease that mainly has bacterial etiology, where immune cells secreting pro-inflammatory cytokines such as IL-1, TNF, and IL-33, which contribute to tissue damage, are invoked [21,44]. The destruction of tissue occurs through stimulation of enzyme production, an effect that may be associated with autoimmune diseases. Therefore, periodontitis and autoimmune diseases share many pathological aspects, including IL-1 and IL-33 production.

IL-33, also called “alarmin”, is part of the IL-1 family and is an instant warning signal of cell damage. [44] In autoimmune or inflammatory processes [25,26,43], IL-33 is secreted by fibroblasts and endothelial cells [45]. IL-33 augments the effect of IgE on secretion of histamine from mast cells and basophils [44,46] by “priming” them [47]. We recently showed that stimulation of human MCs by SP, given together with IL-33, markedly increases secretion and gene expression of the pro-inflammatory cytokines, TNF and IL-1b [48,49].

IL-33 is a new member of the IL-1 family, which regulates innate and adaptive immune systems, promoting inflammatory responses [6]. IL-33 is mainly expressed by keratinocytes, epithelial and endothelial cells [50], and human monocytes [51] and mouse astrocytes [52]. IL-33 acts as an alarmin against injury-induced stress, pathogens, or cell death by activating local immune cells [53,54].

As previously mentioned, both IL-1 alpha and IL-1 beta are found in proform, and therefore, to be mature, they need proteolytic cleavage and NLRP3 [27,28,29,30].

IL-33 proform measures approximately 30 KDa and is processed without the intervention of the inflammasome, while the processing of caspase-1 causes the inactivation of IL-33, rather than providing mature forms of IL33 [55]. IL-1 proform is not active [56] and can be cleaved by a protease such as cathepsin G and elastase, generating the active biological form of IL-1 [57,58,59].

Th2 immune cells are activated by IL-33 to produce cytokines, and this activation may also be accompanied by the activation of CD8^+^ cells [60]. Clinical studies have shown that IL-33 induces the activation of lymphoid cells (ILCs) through mTOR activation [61]. ILCs are involved in IgE-mediated allergic and asthmatic processes, they increase in the blood of patients during seasonal pollination, and they are correlated with disease status. ILCs reside in the tissues of the mucosa and gingiva, where they increase significantly during the chronic inflammatory state. IL-33 regulates human β-defensin 2 (hBD2) in keratinocytes facilitating *Staphylococcus aureus* infection in tissues [62], a process in which immune cells, including MCs, are activated [63].

### 3.1. IL-33 Expression

IL-33 and its ST2 receptor are expressed in various cell types, including MCs. IL-33 is a cellular sensor that mediates the pathogenesis of allergic and autoimmune diseases such as rheumatoid arthritis, psoriasis, inflammatory bowel diseases, etc. This cytokine stimulates TNF by activating MCs and is overexpressed in gingival tissues, with periodontitis and bone loss mediated by the increase of the nuclear factor receptor kappa-β ligand (RANKL) [64]. Elevated IL-33 in saliva could cause autoimmunity-related microbial dysbiosis.

Environmental factors, as well as PAMPs, which are molecular patterns associated with pathogens [64], are able to express the cytokine IL-33, activating the inflammatory system. IL-33 can also be induced by bacteria and viruses, as well as through the TLRs present on primary inflammatory cells, including MCs. Furthermore, MC activating antigens can be allergens of which extracellular ATP is the sensor [65,66]. All of these stimulating effects lead to the generation of proinflammatory cytokines in the periodontal tissue [67]. IL-33 and its surface ST2 receptor were upregulated by IFNγ in keratinocytes derived from patients with AD [68]. TNF, but not IL-17, stimulates secretion of IL-33, which induces expression of IL-6, MCP-1, and VEGF [69]. However, it appears that the type of cytokines/chemokines produced by IL-33 may depend on particular tissues, since the extent of and the type of such mediators vary between sensitized skin and asthmatic airways [70].

IL-33 was discovered as a main ligand to the ST2 (IL-1R4) receptor, which is mostly expressed on the surface of epithelial cells, fibroblasts, and MCs [71]. ST2 is the IL-33 receptor, which is found on the cytoplasmic membrane, and it crosses it and enters the cytoplasm. The exposed part on the membrane is the most abundant, while the cytoplasmic part is the soluble form, which acts as a decoy by binding IL-33 [72]. The receptor complex comprises the ST2 and IL-1 receptor accessory protein [73]. The ST2 receptor is generated by various cell types, including MCs. ST2 receptor IL-33 binds to the IL1RAcP co-receptor, which is shared with IL-1 and initiates the MyD88, IRAK, IRAK4, TRAF6, and IKKabg cascade, and this leads to the activation of NF-kB, a process similar to that of IL-1 [74] (Figure 1).

ST2 activation leads to stimulation of the mitogen-activated protein kinase (MAPK) via TNF receptor-associated factor 6 (TRAF6), which can signal the activator protein-1 (AP-1) via c-Jun N-terminal kinases (JNKs). Therefore, TRAF6 can also activate nuclear factor-κB (NF-κB), resulting in its nuclear translocation and pro-inflammatory gene transcription [74]. For example, ST2 activation of the chronic myeloid leukemia cell line (KU812) results in the release of multiple cytokines through the stimulation of NF-κB, JNK, and p38 MAPK, but not ERK1/2; however, IL-13 generation appears not to require JNK or ERK1-2 signals [75]. In in vitro experiments, it has been reported that IL-33 may also be a DNA-binding cytokine [74].

### 3.2. IL-33 in Allergy and Inflammation

Several authors have reported that IL-33 mediates inflammation of the upper and lower airways [76], confirming that this is a crucial cytokine in allergic and inflammatory responses in the entry districts of microorganisms [77]. The IL-33-mediated inflammatory response in the airways is performed with Th-17 and MC activation [78]. IL-33 is therefore implicated in the crosstalk between MCs and smooth muscle cells in human airways [79]. IL-33 levels are significantly increased in asthmatic subjects compared to healthy subjects [80,81,82,83]. IL-33 also increases in bronchial epithelial cells and periodontal disease upon stimulation with microorganisms or other compounds that can cause inflammation [84,85]. Therefore, in most allergic phenomena and in anaphylaxis, there is an increase in IL-33 [86,87].

Many in vivo experiments on mice, in which lung disease has been induced, have shown high levels of IL-33, confirming the importance of this cytokine in lung inflammation [84,88]. IL-33 is a dangerous signal mediator of epithelial cells and is associated with the response of TH2 lymphocytes in allergic diseases, promoting the activation of innate lymphoid cells 2 (ILC2) and involving innate inflammation [89]. IL-33 participates in the enhancement of ILC2 receptor expression and ILC2 activation, with a mechanism yet to be elucidated. Furthermore, when IL-33 is administered intranasally, it provokes an allergic–inflammatory response, probably involving MC activation [85]. This effect is not present in IL-33-deficient mice [90]. Activation of eosinophils and basophils with pollen also leads to an increase in IL-33, an effect that does not occur with the lack of these cells in inflammatory processes [86]. In addition, in the absence of the ST2 receptor of IL-33 in asthmatic-type pulmonary allergy, inflammation is significantly reduced, and Th2-type cytokines are activated [70]. Moreover, also in atopic dermatitis, an inflammatory allergic disease, there is an increase in IL-33 compared to healthy subjects [91,92,93]. This phenomenon is mediated by IL-33, which also stimulates innate lymphoid cells [94]. In some autoimmune diseases such as psoriasis, elevated levels of IL-33 produced by MCs can occur in the psoriatic scabs of the skin with mediation of the inflammatory process [92,94,95]. In fact, the ST2 receptor of IL-33 is expressed on MCs, which, after activation, can produce substances that increase chemotaxis and amplify inflammation [54,96]. Therefore, the MCs are considered real sensors of cell damage that produce the “alarmin” IL-33 [97]. More recently, cultured MCs derived from bone marrow, stimulated by specific ovalbumin and IgE, induced the expression and release of IL-33, which has an autocrine action on the expression of IL-6 and IL-13 [98], cytokines also important in autoimmunity. In rheumatoid arthritis, an autoimmune disease, MCs participate in the production and activation of the cytokines TNF and IL-6. TNF induces the expression of IL-33, which participates in the pathogenesis of the disease. Inhibition of IL-33 leads to a marked improvement in inflammatory pathology, demonstrating the importance of this cytokine in autoimmune diseases. MCs are hemopoietically derived cells, located close to blood vessels and nerves, where they proliferate primarily in response to stem-cell factors (SCF) [99], but also nerve-growth factors (NGF) [100]. MCs are important for allergic reactions, as well as for mastocytosis, mast-cell-activation disorders, and other inflammatory diseases [101]. IL-33 has also been reported to be involved in the maturation of human MCs [102] and promote MC survival [103]. IL-33 promoted the proliferation of mouse MCs independent of c-kit [104]. Nevertheless, IL-33 was reported to cross-activate the SCF c-kit receptor on MCs [105]. Evidently, IL-1RAcP interacts with c-kit constitutively, and IL-33R binds upon stimulation with SCF, leading to cytokine release [106]. Apparently, inhibition of c-kit signaling also blocked human MC release of IL-16 [107], which had been shown to occur selectively without degranulation [108].

IL-33 augmented the effect of IgE and SCF on activation MCs and basophils [46]. IL-33 induced the release of pro-inflammatory cytokines, especially IL-6, without degranulation from bone-marrow-derived MCs (BMCMCs) [44], and enhanced IL-8 production from human cord blood mast cells (hCBMCs) stimulated by IgE/anti-IgE, but without histamine release [103]. IL-33 augmented human MC release of VEGF in response to SP, but not on its own [109]. Moreover, IL-33 production of IL-13, independent of FcεRI stimulation [110], stimulated PGD_2_ but not tryptase release from activated human MCs [111]. IL-33 was also able to prime murine MCs for enhanced activation by IgG immune complexes [104,112] and stimulated MC-dependent neutrophil influx [113,114]. It has long been known that MCs are activated by various substances, including IgE, which crosslinks the FceRI receptor; however, a variety of biological molecules cannot stimulate MCs to produce inflammatory substances [115]. Upon activation with various triggers [116,117,118], MCs immediately release proinflammatory mediators, such as tryptase and histamine. These compounds are released (in seconds), while subsequently (after several hours) MCs release proinflammatory cytokines (IL-1, IL-6, and IL-33), chemokines (CCL2, IL-8, and CXCL8), prostaglandin (PGD2), and leukotrienes (LTC4, LTD4, and LTE4) [119,120,121]. All of these substances participate in chronic autoimmune inflammation. The release of TNF, both from granules and by synthesis via mRNA, leads to the activation of T lymphocytes [122,123]; meanwhile, the generation of IL-6 and TGF-b is fundamental for the synthesis of Th-17 cells [124,125,126]. All of this, together with the synthesis of IL-33, is part of an immuno-inflammatory framework in which MCs play an important role [127,128], contributing to the autoimmune phenomenon [129,130,131,132].

### 3.3. IL-33 in Inflammatory Autoimmune Disease

MCs are capable of processing microbial antigens by intervening in acquired immunity and play a key role in inflamed periodontal tissue by producing IL-33 and other pro-inflammatory cytokines. MCs play a crucial role in allergic pathogenesis and systemic diseases [133,134], producing pro-inflammatory cytokines of the IL-1 family, effects that can be suppressed by IL-37 by forming a complex with extracellular IL-18Rα and IL1R8. In periodontitis, there is a greater expression of pro-inflammatory cytokines, such as the IL-33 produced by MCs, associated with the pathogenesis of periodontal disease.

IL-33 activation mediates inflammation in autoimmune diseases [129]. The ST2 receptor is often found in the serum of patients with inflammatory and allergic diseases, and it is related to the severity of the disease. It has been reported that, in many autoimmune diseases, such as rheumatoid arthritis (RA) [134], systemic lupus erythematosus (SLE) [135], Sjogren’s syndrome [136], Grave’s disease [137], and Inflammatory Bowel Disease (IBD) [138,139,140], where inflammation plays an important role, the number of MCs and blood levels of IL-33 are increased [26]. Therefore, MCs, in addition to being important in periodontal inflammation, both in the acute phase by producing chemical mediators and in the chronic phase by secreting IL-33 and other pro-inflammatory cytokines [141,142], are also relevant in innate and acquired immunity [143,144,145]. In fact, in atopic dermatitis [144] and psoriasis [109], rheumatoid arthritis [134,146], multiple sclerosis [147], and autism [148], MCs can intervene and selectively release pro-inflammatory cytokines [149]. Moreover, in Alzheimer’s disease [150], MCs can be involved in releasing IL-33 into amyloid plaques, favoring inflammatory and degenerative processes [151]. Moreover, incubation of mouse astrocytes with amyloid-β1-42 increased IL-33 expression [149]. In fact, increasing evidence implicates brain inflammation and cytokines in the pathogenesis of Alzheimer’s disease [152,153]. Brain inflammation may be evident in the earlier stages of the disease and may constitute a more reasonable target for drug development [154,155]. Interestingly, IL-33 was also upregulated in astrocytes and peripheral leukocytes of multiple sclerosis (MS) patients [156]. Moreover, the expression of IL-33 protein and IL-33 genes was increased in patients with remitting–relapsing MS [157]. In non-allergic brain inflammation induced in mice, in which MCs play a crucial role, it has been reported that, by using related W/Wv mice lacking in MCs, inflammation is markedly inhibited [158]. This suggests the importance of CDs in inflammatory pathogenesis [159]. These results are interesting in view of the fact that ANK2 was strongly associated with autism [160,161]. Many children with autism are characterized by allergic symptoms [148,162], and the risk of autism is much more common in children with mastocytosis [163]. In fact, autism involves brain inflammation [164,165] and microglia activation [166,167]. Moreover, there is evidence of crosstalk between microglia and MCs [168]. It is interesting that the diseases discussed above worsen with stress [147,169,170,171], and MCs are activated by the corticotropin-releasing hormone (CRH) secreted under stress [172,173].

## 4. Interleukin-37 (IL-37)

**Figure 2:**
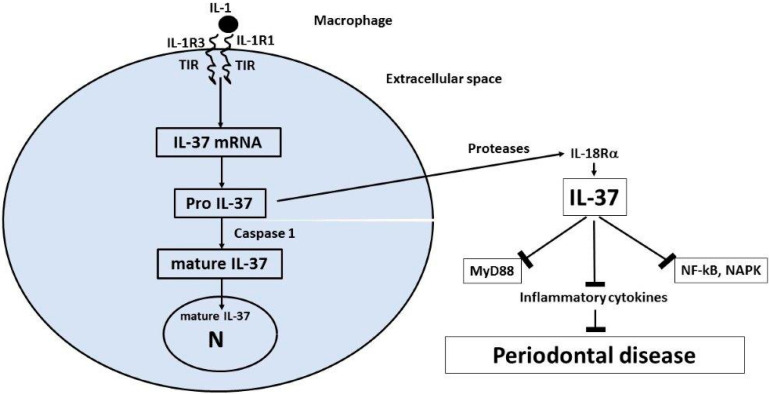
Synthesis, processing, and release of IL-37. Pro-inflammatory stimuli that are TLR agonists, including IL-1, trigger the synthesis of the precursor form of IL-37 (pro-IL-37). Inside the cytoplasm, the precursor (pro-IL-37) is processed by caspase-1 into mature IL-37, and part of it translocates to the nucleus and suppresses inflammatory pathways. At an extracellular level, the proteases may act on pro-IL-37 by transforming it into mature IL-37. Both the immature and mature forms have been reported to be biologically active and may suppress inflammation in periodontal disease [172].

Since IL-37 is a strong blocker of IL-1 and a pro-inflammatory cytokine in periodontal disease, in this review, we speculate that IL-37 treatment of the disease could be a further therapeutic adjunct to traditional medications. IL-37 is a member of the IL-1 family, whose precursor is produced by immune and non-immune cells after an inflammatory process. IL-37 is generated through the activation of a caspase-1, translocates to the nucleus, and inhibits genes involved in inflammation by suppressing the NF-kB and MAPK pathways [58,174,175,176,177] (Figure 2). Monocytic cells and macrophages activated through the TLR produce pro-IL-37, an immature cytokine that is cleaved by caspase-1 and transforms into active mature IL-37, which, in a small amount (20%), enters the nucleus, while the remainder is expelled out of the cell together with the immature form, pro-IL-37, which is also active [178]. MCs that produce extracellular proteases can act on pro-IL-37, transforming it into a more biologically active form, as occurs with IL-37b, which is the most used form in in vitro and rodent experiments [179]. Although no specific receptor for IL-37 has been identified, a number of studies showed that extracellular IL-37 binds to the alpha chain of the IL-18Rα [171,172], but with less affinity than IL-18 [180,181,182]. Binding of IL-37 to the IL-18 receptor and to the decoy receptor 8 (IL-R8) [183,184] causes a strong inhibition of innate immunity [48,179,185], which mediates acute inflammation and autoimmunity [186,187,188], including the pathogenesis of periodontal disease. IL-37 is a protective cytokine against acute and chronic inflammatory diseases; in fact, in autoimmune diseases, the levels of IL-37 are abnormal compared to those of healthy patients. Experimental animals treated with IL-37 show a reduction in inflammatory proteins produced by human monocytic M1 cells, both in vivo and in vitro [185]. It has been reported, in human activated monocytes, that suppression of IL-1-b and IL-6 generation increases the expression of the anti-inflammatory IL-37 cytokine [189,190,191]. Most of the inhibitory effects exerted by IL-37 are currently unknown, so new experiments are awaited to fully clarify this enigma.

## 5. Conclusions

Today, it is known that IL-37 inhibits innate and acquired immunity and, consequently, inflammation [192,193], an effect that could complement the treatment of acute and chronic gingival inflammation, including periodontal disease [194]. The data suggesting that IL-37 acts on the inhibition of mTOR [195], a molecule involved in the stimulation of neurotensin (NT) on human microglia and on the suppression of the inflammasome in mice [196,197], are yet to be confirmed.

Since IL-1 induces IL-33, it is pertinent to think that, by blocking IL-1 with IL-37, there would be an inhibition of inflammation in periodontal diseases; however, these data will need to be confirmed in the future.

In this work, therefore, we hypothesize that IL-37, being a blocker of IL-1, one of the main inflammatory cytokines in the pathogenesis of periodontitis, may be of help in the therapy of this common disease.
